# A Newly Described Bovine Type 2 Scurs Syndrome Segregates with a Frame-Shift Mutation in *TWIST1*

**DOI:** 10.1371/journal.pone.0022242

**Published:** 2011-07-21

**Authors:** Aurélien Capitan, Cécile Grohs, Bernard Weiss, Marie-Noëlle Rossignol, Patrick Reversé, André Eggen

**Affiliations:** 1 Unité Mixte de Recherche 1313 Génétique Animale et Biologie Intégrative, Institut National de la Recherche Agronomique, Jouy-en-Josas, France; 2 Service Génétique, Département Fédéral, Union Nationale des Coopératives d'Elevage et d'Insémination Animale, Paris, France; 3 Laboratoire d'Analyses Génétiques pour les Espèces Animales, Jouy-en-Josas, France; 4 Section Charolaise, Gènes Diffusion, Douai, France; Ohio State University Medical Center, United States of America

## Abstract

The developmental pathways involved in horn development are complex and still poorly understood. Here we report the description of a new dominant inherited syndrome in the bovine Charolais breed that we have named type 2 scurs. Clinical examination revealed that, despite a strong phenotypic variability, all affected individuals show both horn abnormalities similar to classical scurs phenotype and skull interfrontal suture synostosis. Based on a genome-wide linkage analysis using Illumina BovineSNP50 BeadChip genotyping data from 57 half-sib and full-sib progeny, this locus was mapped to a 1.7 Mb interval on bovine chromosome 4. Within this region, the *TWIST1* gene encoding a transcription factor was considered as a strong candidate gene since its haploinsufficiency is responsible for the human Saethre-Chotzen syndrome, characterized by skull coronal suture synostosis. Sequencing of the *TWIST1* gene identified a *c.148_157dup* (*p.A56RfsX87*) frame-shift mutation predicted to completely inactivate this gene. Genotyping 17 scurred and 20 horned founders of our pedigree as well as 48 unrelated horned controls revealed a perfect association between this mutation and the type 2 scurs phenotype. Subsequent genotyping of 32 individuals born from heterozygous parents showed that homozygous mutated progeny are completely absent, which is consistent with the embryonic lethality reported in *Drosophila* and mouse suffering from TWIST1 complete insufficiency. Finally, data from previous studies on model species and a fine description of type 2 scurs symptoms allowed us to propose different mechanisms to explain the features of this syndrome. In conclusion, this first report on the identification of a potential causal mutation affecting horn development in cattle offers a unique opportunity to better understand horn ontogenesis.

## Introduction

Horns in bovine as in all members of the Cavicorn superfamily, are permanent and not ramified. They consist of a bony core covered by a corium producing the keratin sheath. Contrary to antlers in deer, the developmental pathways involved in horn formation have not been extensively studied and are still poorly understood.

Studies by Dove [1] contributed greatly to the comprehension of this complex process. Using tissue transplantation, Dove showed that: (i) the bony core is not an outgrowth of the skull but originates from a separated center of ossification located in the dermis and hypodermis of the calves' horn bud; (ii) the keratinization of the horn bud epidermis does not induce ossification of the underlying dermis and hypodermis and conversely, thus both phenomena are probably programmed during embryogenesis; (iii) the ossifying hypodermal tissue induces the frontal bone to grow upward and to form the base of the horn spike, then it fuses with the skull by dissolving it locally. (Figure S1). Thus, horn development is the result of the differentiation and remodeling of various tissues originating from two distinct germ layers: ectoderm and mesoderm.

Genetic abnormalities affecting horn development represent unique models to identify genes and pathways involved in this process. Two main approaches are generally used to achieve this goal: comparison between wild-type and affected horn buds gene expression (as recently used by Mariasegaram et al. [2]) or genetic mapping followed by candidate gene sequencing to identify the causal mutation. In this study, the latter approach was used to determine the genetic basis of the polled and scurs phenotypes in the French Charolais breed.

The polled phenotype is characterized by the complete absence of horns as well as of any type of corneous growth. On the contrary, scurs share similar shapes and locations with horns but they are generally smaller and characterized by an absence of fusion between the bony core and the skull [1], [3], [4]. Even if several exceptions have been reported (for a review see [5]), it is generally believed that the genetic determinism of these horn abnormalities involves the interaction of two autosomal biallelic loci: the *polled* and *scurs* loci. Indeed, the P allele of the *polled* locus is dominant and specifies the absence of wild type horns whereas the presence of scurs or the complete absence of appendage is determined by the Sc and sc alleles of the *scurs* locus, respectively [6]–[8]. Numerous studies have mapped the *polled* locus to the centromeric region of BTA01 in various breeds, but to date the causal mutation has not been identified and/or published [9]–[14]. However, only one study mapped the *scurs* locus on BTA19 in a crossbred pedigree [15] and we were not able to confirm this result in the French Charolais breed as reported in a previous study based on BTA19 microsatellites genotyping data [5].

In order to fine-map both loci, we performed Illumina BovineSNP50 genotyping on a French Charolais pedigree consisting of 323 individuals (73 horned, 153 scurred and 97 polled) representing 40 paternal and 35 maternal half-sib families (unpublished data). After haplotype reconstruction for the BTA01 centromeric region, two different haplotypes were identified among the polled individuals but absent among the horned individuals. To avoid potential bias due to different interactions between the scurs locus and two different polled mutations, we classified the polled and scurred individuals into two groups, according to their polled haplotype at BTA01, before performing the mapping of the scurs locus within each group. Interestingly, several scurred individuals could not be classified into these two groups. In other words, those animals were scurred without exhibiting one of the two identified polled haplotypes on BTA01. A pedigree analysis revealed that these animals are related to the same sire over a maximum of six generations and that the scurs phenotype is transmitted in a pattern consistent with autosomal dominant inheritance. However this transmission occured independently from the BTA01 haplotype pointing to a different etiology than the common scurred phenotype, the expression of which is fully dependent on the presence of the P allele from the *polled* locus [7], [8]. Based on these evidences, this new genetic disorder affecting horn development was called type 2 scurs.

In the study reported here, our objective was (i) to describe more precisely the type 2 scurs phenotype and (ii) to fine-map this locus and identify the causal mutation, in order to better understand the developmental pathways involved in bovine horn formation.

## Results

### Clinical findings

**Figure 1:**
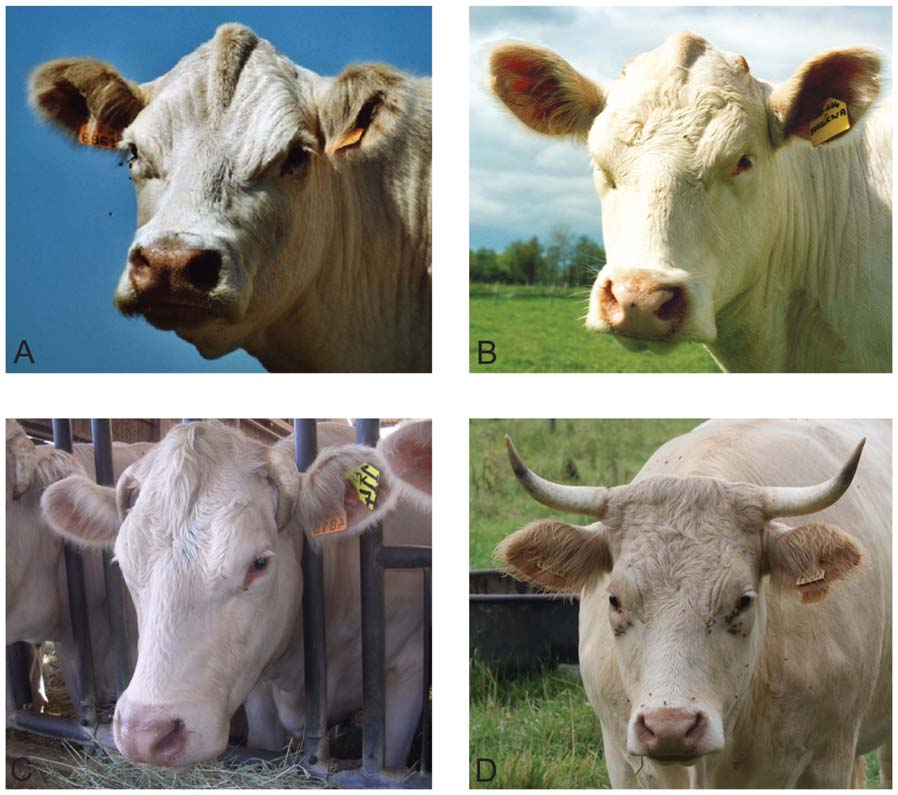
Phenotypic description of type 2 scurs syndrome. Ten-(A), two-(B) and four-(C) year old females affected by type 2 scurs syndrome. Note the marked phenotypic variability and the negative correlation between the size of scurs and the importance of acrocephaly and ridge-shaped extra bone deposition along the interfrontal suture. (D). Four-year old horned female.

**Figure 2:**
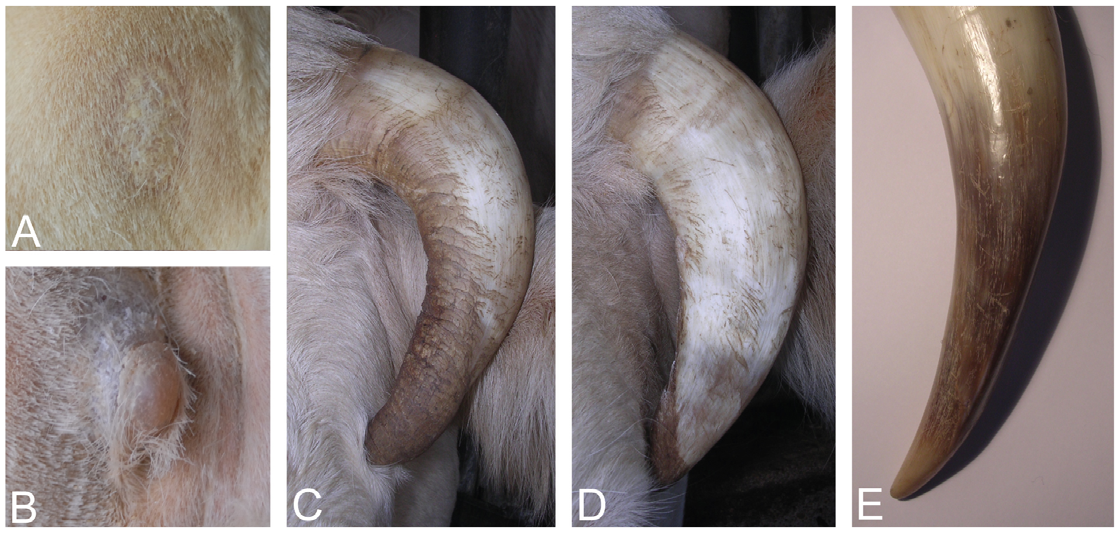
Details of the type 2 scurs keratin sheath. (A). Scaly patches. (B). Small scurs (∼2 cm) with irregular keratin sheath. (C and D). Long scurs (∼15 cm) with an irregular keratin sheath at their end (see arrows). (E). End of a normal horn (∼25 cm) with a regular keratin sheath.

**Figure 3:**
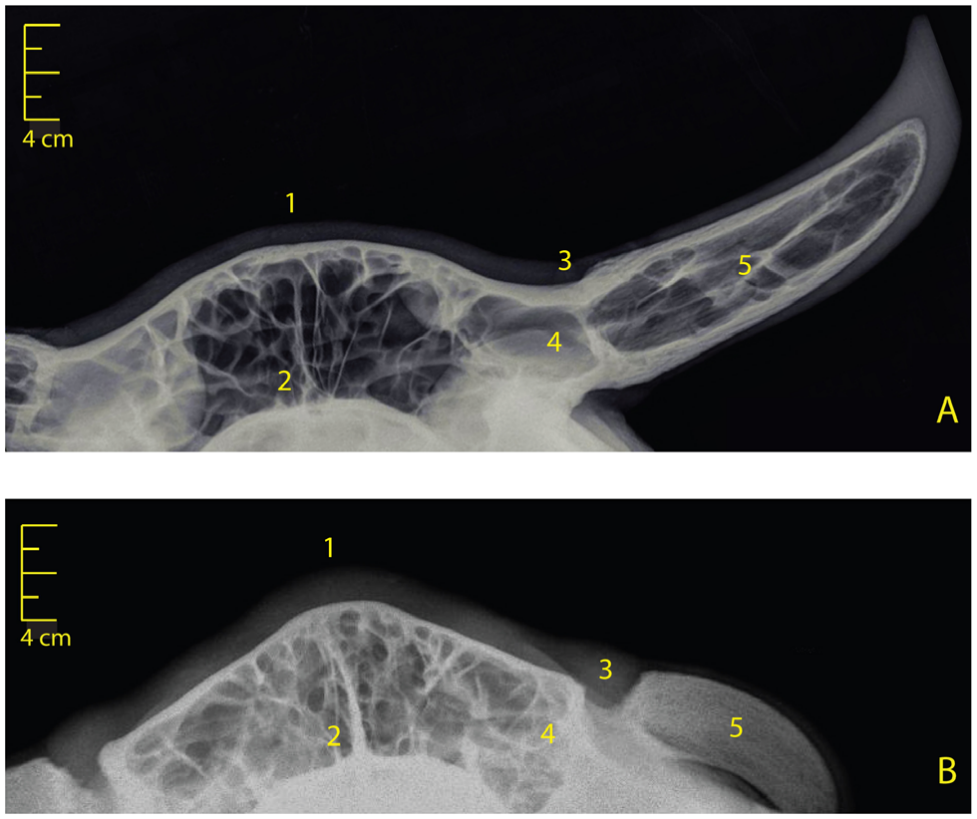
Frontal radiographs of type 2 scurred and horned skulls. (A). Control skull of a five-year old horned female. (B). Skull of a four-year old female affected by the type 2 scurs and carrying long scurs. Note: (1) the particular shape of the poll, (2) the denser calcification of the interfrontal suture, (3) the absence of fusion between the frontal bone and the horn bony core, (4) the absence of frontal bone drawing up and (5) the non-pneumatization of the bony core in affected vs. horned skulls.

Visual examinations showed a strong phenotypic variability between individuals and genders among affected animals:

- The size of scurs varies from small scabs to 15 cm-long appendages in adult females (Figure 1) whereas in adult males scurs are systematically massive (more than 10 cm-long) and often less mobile, but still not completely fused to the skull. Furthermore, scurs grow earlier in life in males: they are usually detected at first examination (between 4 and 6 months) unlike in females where they become visible at second examination (between 9 and 18 months) or later.
- The structure of the keratin sheath also presents different levels of alteration depending on the scurs size: tiny scurs develop as scabs made of scaly patches, whereas small scurs and the terminal portion of long scurs are covered by irregular keratin sheets (Figure 2).
- In addition, affected individuals show mild to pronounced acrocephaly and a ridge-shaped extra bone deposition along the interfrontal suture, which both appear to be negatively correlated with the size of scurs (Figure 1). These pathologies are also attested by the particular shape of the poll and the denser calcification of the interfrontal suture of the type 2 scurred vs horned skull radiographs (Figure 3).

Radiographs also revealed that (i) the frontal bone of affected individuals is not drawn up to form the basilar portion of the horn spike (ii) the scurs bony core is not pneumatized and (iii) the space between the skull and the bony core is filled with soft tissues.

In summary, despite a strong phenotypic variability, all affected individuals show both horn abnormalities similar to classical scurs phenotype and skull interfrontal suture synostosis, constituting a single pathological entity (*i.e.* a syndrome). These animals are readily distinguishable from their wild-type relatives and from the wild-type controls: (i) horns of wild-type male and female born from affected dams are always visible at first examination and often already fused to the skull at this time; (ii) in adults they are firmly attached to the skull, their keratin sheath is regular and their size is longer than 15 cm; (iii) finally none of the wild-type animals show evidence of acrocephaly and extra bone deposition along the skull interfrontal suture.

### Mapping of the type 2 scurs gene

**Figure 4:**
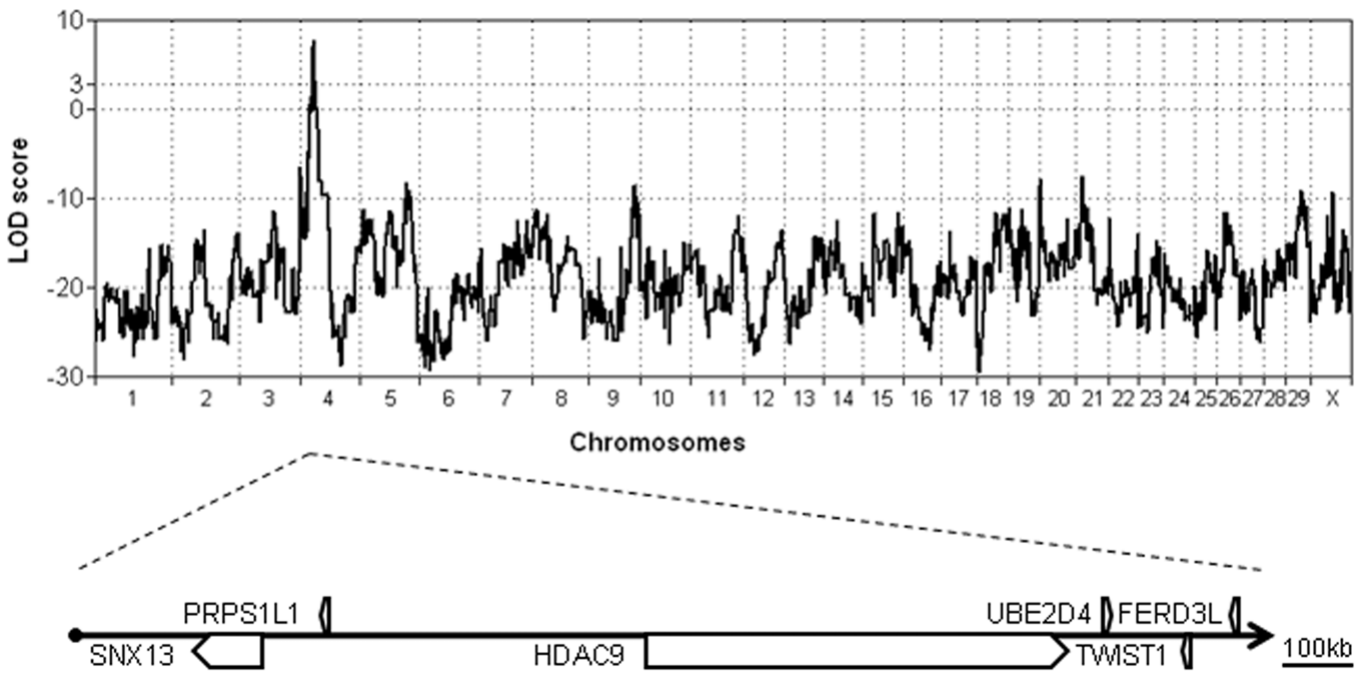
Genome-wide multipoint linkage analysis of type 2 scurs syndrome. The genome-wide scan reveals a significant linkage (maximum LOD score = 7.2) with clusters of markers located on chromosome 4. The 95% confidence interval spans 1.7 Mb encompassing six different genes.

As shown in Figure 4, the genome-wide scan revealed a significant linkage (maximum LOD score of 7.2) between the type 2 scurs phenotype and several clusters of markers located on chromosome 4. The 95% confidence interval spanned 1.7 Mb (from marker ARS-BFGL-NGS-57582 to BTB-01114634) encompassing six different genes: *SNX13*, *PRPS1L1*, *HDAC9*, *UBE2D4*, *TWIST1* and *FERD3L*. Among them *TWIST1* was the most compelling candidate gene since it encodes a basic helix-loop-helix (bHLH) transcription factor regulating many processes including cranial suture patterning and fusion [16], [17]. Numerous mutations in this gene have been reported to be responsible for the human Saethre-Chotzen syndrome (SCS; OMIM#101400) also known as Acrocephalosyndactyly type III. The hallmark of this autosomal-dominant syndrome is acrocephaly (due to premature fusion –synostosis– of the skull coronal suture) associated with variable additional features such as mild craniofacial and limb deformities (for a review see [18], [19]).

### Mutation analysis

Sequencing the entire *TWIST1* gene in a trio consisting of two affected females and one unaffected male allowed us to identify a 10-bp duplication (*c.148_157dup*) in a GC-rich fragment of exon 1. Subsequent genotyping of this mutation on a broader panel of animals revealed a perfect association with the type 2 scurs phenotype: all 17 affected founders of our pedigree were heterozygous whereas all 20 non-affected founders and 48 unrelated controls were homozygous for the wild-type allele.

### Assumed consequence of the *TWIST1 c.148_157dup* mutation

**Figure 5:**
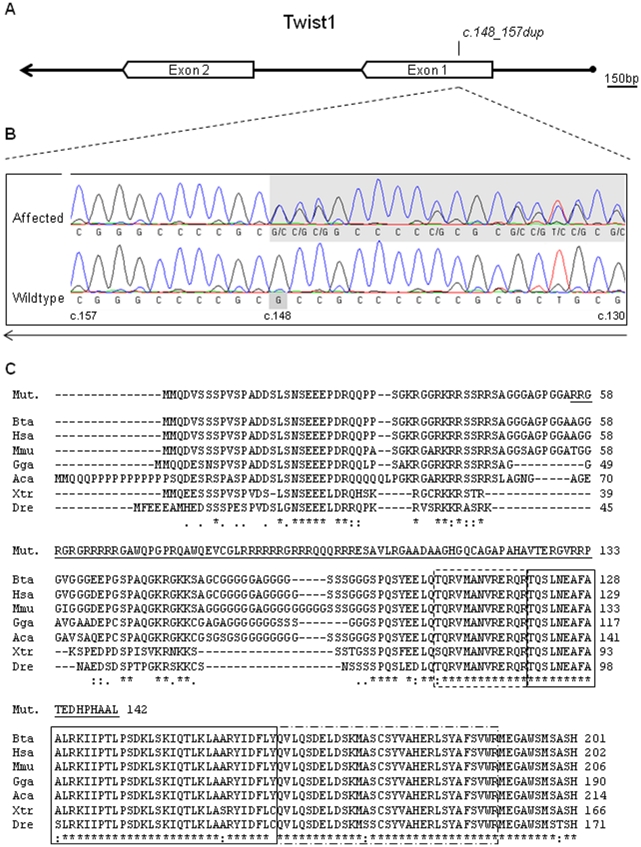
Characterization of the *TWIST1 c.148_157dup* mutation. (A) *TWIST1* gene's organization scheme; (B) DNA sequencing chromatograms showing the *c.148_157dup* mutation of type 2 scurs affected animals and the wild type allele; (C) putative *p.A56RfsX87* mutated protein and multispecies alignment of the TWIST1 protein sequence using CLUSTALW [21]. Cattle (Bta), human (Hsa), mouse (Mmu), chicken (Gga), anolis (Aca), xenopus (Xtr) and zebrafish (Dre) sequences accession numbers are respectively DAA30767, NP_000465, AAH33434, NP_990070, DAA06059, AAH74558, ABC73066 in Genbank. The frame-shift change in the bovine p.A56RfsX87 mutated protein (Mut.) is underlined. The basic, helix-loop-helix and tryptophan–arginine domains are respectively highlighted with dashed, solid and mixed dashed boxes [22].

*TWIST1* has two highly conserved domains: the basic helix–loop–helix (bHLH) domain and the tryptophan–arginine (WR) domain [20] (Figure 5). The first domain is a bipartite domain for DNA binding (basic motif) and protein-protein interactions (helix-loop-helix motif) shared by numerous transcription factors [23]–[26]. The second one, also known as the TWIST box since it is specific to this subfamily, has been shown to inhibit the function of the Runx2 DNA binding domain [27] and to be essential for the transactivating function of TWIST1 in mice [28].

As shown in Figure 5, the *c.148_157dup* mutation is predicted to cause a frame-shift change of *TWIST1* with Alanine-56 as the first affected amino acid (*p.A56RfsX87*). This frame-shift is assumed to completely inactivate this gene because the 142-amino acid mutated protein lacks both above mentioned functional domains.

Since targeted twist-null mutations are embryonic lethal in *Drosophila* [29], [30] and mouse [31], we genotyped 32 individuals born from heterozygous parents to look for distorted Mendelian transmission. As presented in Table 1, no homozygous individual for the *c.148_157dup* was observed. Moreover the observed genotype distribution fitted its expectation under the hypothesis of lethality of this mutation in the homozygous state. Thus, we conclude that the *TWIST1 c.148_157dup* mutation associated with type 2 scurs may be homozygous lethal.

**Table 1:** Results of *TWIST1 c.148_157dup* mutation genotyping in 32 progeny born from heterozygous parents.

Genotypes	Homozygous wild-type	Heterozygous	Homozygous *c.148_157dup*
Expected distribution if *c.148_157dup* is **not lethal** in the homozygous state	8	16	8
Expected distribution if *c.148_157dup* is **lethal** in the homozygous state	10.66	21.33	0
Observed distribution	10	22	0

## Discussion

In this study, we describe the identification, the characterization and the fine-mapping of a new genetic defect affecting both horn development and skull interfrontal suture fusion in cattle.

This new syndrome was named type 2 scurs for its striking similarity with the already known scurs phenotype. In both syndromes, horn appendages are indistinguishable at the macroscopic scale: (i) the frontal bone is not drawn up to form the basilar portion of the horn spike; (ii) the space between the skull and the bony core is filled with soft tissues; (iii) the bony core is densely ossified and covered by an irregular keratin sheath, and finally (iv) there is a marked phenotypic variability between individual and gender [1], [3]. Such similarity suggests a close etiology between these syndromes: genes involved in the same metabolic pathway might be responsible for these abnormalities.

Our study shows that the similarity between these two disorders could have interfered with the identification of the genetic determinism of the “classical” scurs and the type 2 scurs, explaining the numerous exceptions [5] reported to the scurs genetic determinism proposed by Long and Gregory [7] and Brem et al. [8].

In addition, we identified a frameshift mutation (*p.A56RfsX87*) predicted to inactivate *TWIST1* and demonstrated that a perfect association exists between this mutation and the type 2 scurs syndrome.

Although we cannot provide functional proof of the causality of the *p.A56RfsX87* mutation at this time, the large amount of functional data available for the *TWIST1* gene strongly supports this hypothesis.

Indeed, more than 80 mutations in the *TWIST1* gene have been reported in humans as the cause of skull coronal suture synostosis, a symptom of the Saethre-Chotzen syndrome [18], [19], [32]–[36]. Functional studies have identified TWIST1 haploinsufficiency as the disease-causing mechanism of this syndrome and unravelled the major role played by this gene in the regulation of cranial suture patterning and fusion [16], [17], [27], [37]–[43]. Namely TWIST1 deficiency inhibits osteogenic stem cells proliferation and leads to premature osteoblast differentiation altering the balance between these two phenomena which is essential for normal sutural growth [44]. Therefore, we assume that the *p.A56RfsX87* mutation causes TWIST1 haploinsufficiency which in turn is responsible for the skull interfrontal suture synostosis observed in cattle affected by the type 2 scurs syndrome.

Moreover the absence of homozygous individuals for the mutation in our second pedigree is consistent with the embryonic lethality reported in *Drosophila* and mouse suffering from TWIST1 complete insufficiency [29]–[31]. Contrary to the *Drosophila* mutant, *twist1 -/-* mouse embryos undergo normal gastrulation. However, later they display severe defects in cranial neural tube closure, head mesenchyme, somites and limb buds and finally die at E10.5–11 [31]. These experiments have underlined the critical role played by TWIST1 in diverse developmental pathways during embryogenesis such as specification of the mesodermal somites' derivatives and neural crest cell migration and differentiation (for a review see [45]–[48]). Thus, we believe that homozygosity for the bovine *p.A56RfsX87* mutation causes TWIST1 complete insufficiency and is embryonic lethal.

Since all available model animals are not horned, it is not possible to infer from previous studies the possible mode of action of the *TWIST1 p.A56RfsX87* mutation on the development of scurs. However, fine examination of the affected animals reveals interesting clues on the underlying mechanisms.

As reported in the human Saethre-Chotzen syndrome, there is a marked phenotypic variability (including horn development) among individuals carrying the same *TWIST1* mutation. Moreover, there is a positive correlation between the level of horn abnormality and the importance of craniosynostosis symptoms. These facts suggest that the mechanism causing the scurs phenotype is the same than for craniosynostosis, *i.e.* TWIST1 haploinsufficiency. Nevertheless contrary to craniosynostosis, the antiosteogenic function of TWIST1 cannot be the only cause of horn abnormalities as attested hereafter.

Despite the marked phenotypic variability, all *p.A56RfsX87/+* animals present both bony core and keratin sheath abnormalities. Since the keratinization of the normal horn bud epidermis and the ossification of the underlying dermis and hypodermis are both programmed during embryogenesis and not induced by each other after birth [1], this observation rules out a possible action of an abnormal bony core on the organization of the overlying corium. Rather, it advocates for three possible etiologies: (i) an early role of TWIST1 in horn bud cells programming during embryogenesis or fetal life; (ii) postnatal requirement of TWIST1 for the modification of normally programmed horn bud epidermis and underlying tissues in the corium and bony core respectively; or (iii) both.

The first hypothesis is consistent with the above-mentioned critical role played by TWIST1 in cell lineage specification and differentiation during embryogenesis. The second is supported by the unique role played by TWIST1 in promoting cell dedifferentiation, migration and proliferation in processes like cancer or Epithelial-Mesenchymal Transition (EMT) [49], [50]. Interestingly, Mariasegaram et al. [2] have observed a marked enrichment of gene networks relating to EMT by studying the differentially expressed genes between horn buds from 1 to 2 week-old polled, scurred and horned calves using a bovine gene expression microarray. However, they did not report a significant difference in *TWIST1* expression between the three categories. This result suggests an earlier involvement of TWIST1 in processes leading to epidermis keratinisation and bony core ossification (hypothesis 1).

In conclusion, we describe a new autosomal dominant inherited syndrome characterised by horn development anomalies, craniosynostosis and an absence of homozygous affected calves. In addition, we have identified the *p.A56RfsX87* mutation in the *TWIST1* gene as the candidate causative mutation and propose different mechanisms involving TWIST1 haploinsufficiency in diverse developmental pathways to explain the three main features of this syndrome. To our knowledge, this is the first report on the identification of a potential causal mutation affecting horn development in cattle. Better, among Bovinae, type 2 scurs would be the only genetic disorder affecting horn development explained by a simple mechanism possibly involving loss of gene function. This makes type 2 scurs an ideal model to study horn ontogenesis. The detailed involvement of TWIST1 in horn development remains to be investigated by functional studies.

## Materials and Methods

### Ethics statement

Experiments reported in this work comply with the French National Institute for Agricultural Research (INRA) ethical guidelines. Animals were extremely well cared. Approval by the INRA Ethical Committee was not necessary for blood sampling, sperm sampling, radiographs and routine husbandry procedures. Blood was collected by the following agricultural technicians licensed by the French *Etablissements Départementaux de l'Elevage (EDE)*: Rémi Bierbaum, Christophe Caron, Vincent Colas, Michel Dewaele, Bruno Elmanowsky, Denis Faradji, Sébastien Landemaine, Jean-Marie Moinel, Arnaud Poilvert, Bernard Raimbault, Stéphane Thibaux and Arnaud Tranier. Sperm was obtained from semen straws generously provided by Gènes Diffusion and UCATRC breeding companies. Radiographs were performed by Dominique Rémy and Guillaume Belbis (licensed veterinarians). All the samples were obtained with the permission of the French Polled Charolais Program.

### Animals

Seventeen scurred dams, 20 horned Artificial Insemination (AI) sires and their 57 progeny (40 scurred and 17 horned) were genotyped for linkage analysis. These individuals belong to the French Polled Charolais Program which aims at producing high genetic value polled sires by mating the best horned AI sires to polled and scurred cows for several generations. All the scurred dams are related to the same sire over a maximum of five generations.

Sequencing of the candidate gene was performed on a trio consisting of a scurred dam, a horned bull and the scurred heifer. In addition, 48 unrelated horned Charolais were recruited as controls. Finally, 32 additional individuals born from heterozygous parents (five sires and 15 dams) were used to study the Mendelian transmission of the candidate mutation.

### Phenotypes

The progeny were phenotyped twice as described in Capitan et al. [5] whereas founders and control individuals were phenotyped once at adulthood. All types of corneous growths that were loosely attached to the skull were considered as scurs [1], [3]–[5]. Following the identification of type 2 scurs, most of the affected individuals were re-examined by visual inspection to refine the phenotype and to search for other associated abnormalities. To complete this study, the skulls of a four-year old female carrying long scurs and a five-year old horned control were radiographed using a Gierth HF 80 Plus (Vtrade international, Fernelmont, Belgium) with the following parameters: 50 volts, 10 mA and 80 cm. Finally, a survey was carried out among breeders to collect all past observations noted among such animals and their ancestors.

### Samples

DNA was extracted from blood using the Wizard® Genomic DNA purification Kit (Promega, Charbonnières-les-bains, France) or from sperm using a standard phenol-chloroform method.

### Linkage analysis

DNA samples were genotyped with the Illumina BovineSNP50 BeadChip [51]. Marker order and map distances were based on the bovine sequence assembly Btau_4.0, assuming 1 Mb for 1 cM. A genome-wide multipoint linkage analysis was performed using MERLIN software (version 1.1.2) [52] and assuming a dominant model of disease inheritance with an allele frequency of 0.0001. Penetrance values were set at 0.01, 0.99 and 0.99 for the homozygous wild-type, heterozygous and homozygous affected individuals, respectively. The marker-marker linkage disequilibrium was modeled using the – rsq 0.1 option. Finally, genotypes for BTAX markers of the bull-calves sires were set as missing to avoid Mendelian errors.

### Mutation analysis

PCR primers covering the whole *TWIST1* gene were designed from the bovine genome sequence assembly Btau_4.0 with Primer3 sofware [53] (Table S1). PCR reactions were performed using the Go-Taq Flexi (Promega, Charbonnières-les-bains, France) or the GC-RICH PCR System (Roche, Meylan, France) according to the manufacturer's instructions on a PTC-100 thermocycler (BioRad, Marnes-la-Coquette, France). The resulting amplicons were purified on MultiScreen PCR96 Filter Plates (Millipore, Molsheim, France) and bidirectionnally sequenced by Qiagen (Hilden, Germany) using conventional Sanger sequencing. Polymorphism was detected with the NovoSNP software [54]. The candidate mutation was subsequently genotyped by PCR-sequencing of exon 1 under the same conditions.

## Supporting Information

Figure S1 **Horn development stages adapted from Dove's (1935) experimental report.**
(PDF)

Table S1 ***TWIST1*** **primers sequences.**
(PDF)
