# Supplementary figures and images for: A Newly Described Bovine Type 2 Scurs Syndrome Segregates with a Frame-Shift Mutation in TWIST1
Source: PLoS One. 2011 Jul 21;6(7):e22242. doi: 10.1371/journal.pone.0022242 (PMC3141036; doi:10.1371/journal.pone.0022242)

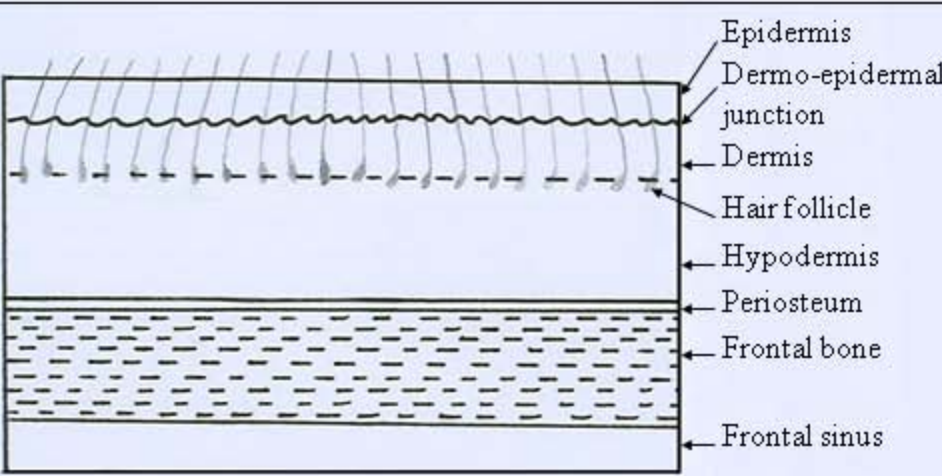

At birth

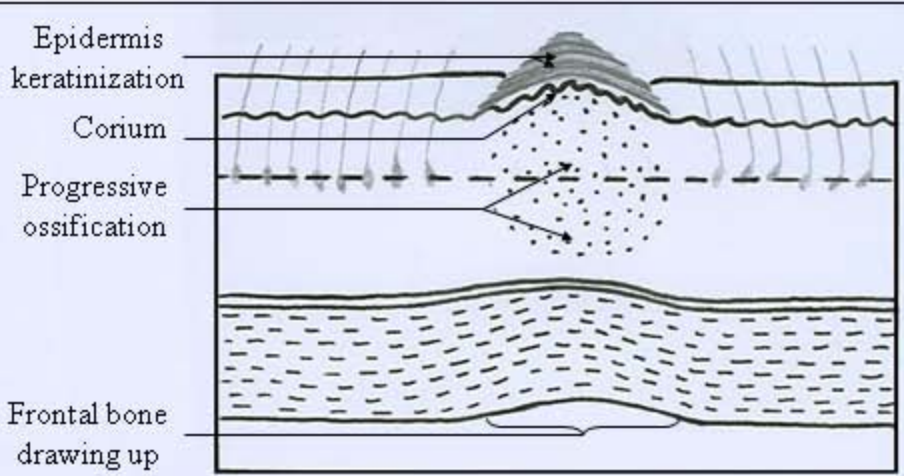

Few weeks after birth

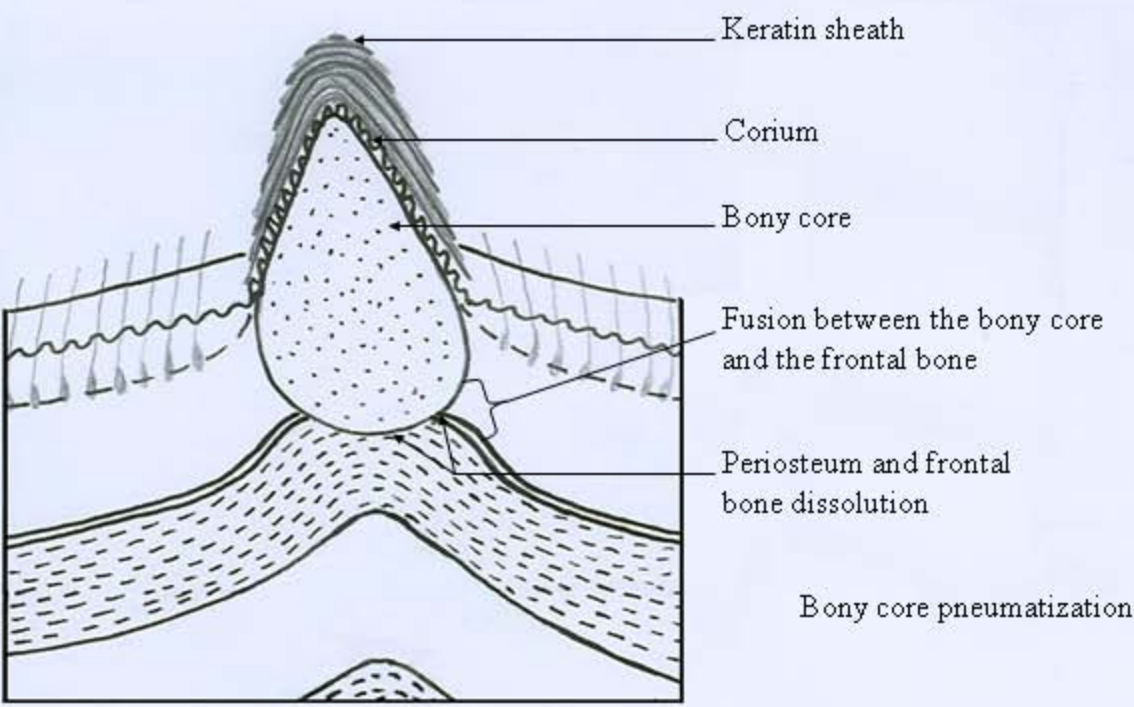

Between 4 and 6 months of age

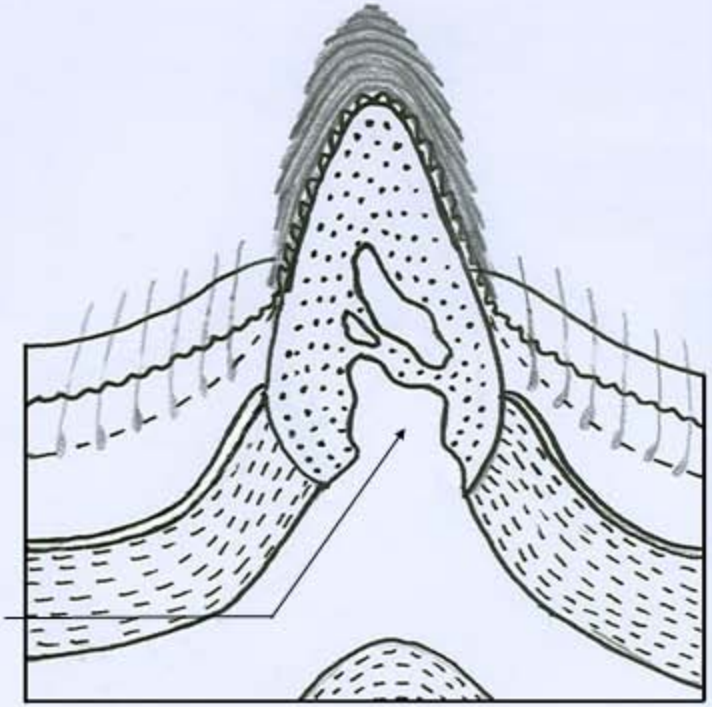

After 6 months of age

Supplement: Figure S1 — Horn development stages adapted from Dove's (1935) experimental report. (PDF) [file pone.0022242.s001.pdf]
