# Supplementary material for: A Newly Described Bovine Type 2 Scurs Syndrome Segregates with a Frame-Shift Mutation in TWIST1
Source: PLoS One. 2011 Jul 21;6(7):e22242. doi: 10.1371/journal.pone.0022242 (PMC3141036; doi:10.1371/journal.pone.0022242)
Supplement: Table S1 — TWIST1 primers sequences. (PDF) [file pone.0022242.s002.pdf]

**Primer names**	**Sequences**
TWIST1_5UTRF	CCCCGAGGTCCAAAAAGAA
TWIST1_5UTRR	GAGCCGACATTTACAGTCATAACG
TWIST1_Ex1F	CTCGTCGCTCTGTAGGACCTG
TWIST1_Ex1R	AGGTATAAGAGCCTCCAAGTCTGC
TWIST1_Int1F	GTTTCTTTGAATTTGGGTTCTGCT
TWIST1_Int1R	GCCAGGTACATCGACTTCCTCTAC
TWIST1_Ex2F	TCCTGGGTGTCTCCAGAGTT
TWIST1_Ex2R	CAGCGCCTCTCTTTCTTTTG
TWIST1_3UTRF	TGGCCCAGAAATAACTAGAATGA
TWIST1_3UTRR	TGACCCATGGTAAAATGCAA
